# The Tomato and Cancer

**Published:** 1890-12-13

**Authors:** W. T. Fernie


### The Tomato and Cancer.

By W. T. Fernie, M.D.

In the year of grace 1836 a memorable letter attributed to Mr. Pickwick, and read at his famous trial with damaging effect, by Serjeant Buzfuz, ran thus: "Dear Mrs. B.,—Chops, and tomato sauce.—Yours, Pickwick." From this it is evident that, fifty or more years ago, some knowledge of the Tomato, or Love Apple, as a delicacy at table, obtained amongst epicures. But not until much more recently has the ruddy, polished, handsome fruit found its way into our greengrocers' shops, and become an article of popular consumption. Introduced at first from South America to the tables of the wealthy, it remained for a while an exclusive luxury grown by the rich, like pine-apples and melons. Since that time the tomato has steadily acquired an increasing popularity with the middle classes; and during the present year its use has become common even among our working people. Cheap tomatoes by barrowloads have lately found a ready sale in the thoroughfares of London and of other large towns, being vended when most plentiful at as low a price as twopence the pound. To meet this modern demand large crops of the profitable fruit are now produced in France, chiefly at Bordeaux, and in the Channel Islands, from which places most of the commoner sort is imported. Much of the favour which has attached itself to the tomato is due to a widespread impression that it is good for the liver and a preventive of biliousness. At the same time, a consensus of opinion has prevailed generally as to the wholesome merits and the refreshing qualities of this picturesque fruit. But just lately certain lurking misgivings have occupied the minds of not a few persons lest the brilliant esculent, which has attained such a high position in public esteem, should after all be an enemy in painted disguise. Rumours have gone abroad that habitual tomato-eaters are especially liable to the destructive inroads of cancerous disease. Therefore a short inquiry into the true character and the actual properties of the tomato may be regarded as useful and opportune.

Closely allied to the potato, and belonging to the same botanical genus of Solanums (which include also the deadly Nightshade and the Henbane), the tomato—Lycopersicum—is a plant of Mexican, or American Indian, origin. Its brightly-coloured fruit was first introduced as "Mala Œthiopica," or the Apples of the Moors, and they bore the Italian designation of Pomi dei Mori. This name became presently corrupted in the French to "pommes d'amour;" thence in English to the epithet "Love Apples," a perversion which shows by what curious methods primary names may become strangely and incongruously changed. The term Lycopersicum bestowed on the tomato signifies a wolf's peach, because some parts of the plant are thought to excite the animal passions. Parkinson says it was originally called Tumatle by the South Americans. The best fruit is supposed to grow within sight, or smell, of the sea. Gerarde tells us that "'Apples of Love' yield very little nourishment to the body, and the same is naught and corrupt; also, their moisture is in this country flashy and insipid, for want of the sufficient heat of the sun in their ripening." There was an unusually large crop produced abroad last year, and hence the recent cheapness of tomatoes in our streets. Green when young, they acquire a bright yellow hue before reaching maturity, being now like "apples of gold in pictures of silver," as described in the Book of Proverbs. When ripe they are smooth, shining, furrowed, and of a beautiful red. Chemically, the Love Apple, like its namesake of our English orchards, contains citric and malic acids. It further possesses oxalic acid, in common with the sorrel of our fields and the rhubarb of our kitchen gardens, on which account each of this vegetable triad is unsuitable for gouty constitutions disposed to the formation of oxalate of lime in the blood. With such persons a single free indulgence in tomatoes may provoke a sharp attack of gout, with the abundant passage in the urine of oxalate of lime as dumb-bell crystals. Otherwise there are strong reasons for supposing that the tomato is a salubrious fruit, of special purifying value. Dr. King Chambers, physician to the Prince of Wales, classifies it among remedies against scurvy, and adds that brown bread, mixed with tomatoes, makes a capital sauce for costive persons. The fruit owns a singular property in connection with diseases of plants, which certainly suggests its probable worth as a protector against bacterial germs and microbes in our bodies, when taken as food. If a tomato shrub is uprooted at the end of the summer and allowed to wither on the bough of a fruit tree, or if it is burnt beneath this fruit tree, it will not only kill any blight which may be present, but will also preserve the tree against any future invasion by blight. The hostility thus evinced by the plant to low organisms is due to the presence of sulphur, which the tomato shrub largely contains, and which is rendered up in an active state by decay or by burning. Now, remembering that digestion likewise splits up the tomato into its chemical constituents, and releases its sulphur within us, we may fairly assume that persons who eat tomatoes habitually are likely to have a particular immunity from bacterial and putrefactive diseases, and we are justified in thinking it altogether improbable that tomatoes will develop cancer, which is essentially a disease of vitiated blood and of degenerate cell-tissue. Vegetarians who eat tomatoes constantly and freely, claim that cancer is a disease almost unknown among their ranks. Dr. Allinson, a champion of this creed, rather attributes the dire malady to the excessive eating of butcher's meat; whilst an Italian doctor, writing from Rome, gives it as the experience of himself and of his medical brethren that cancer is as common in Italy and Sicily among vegetarians as among mixed eaters. Lately, in England, Mr. Lawson Tait has been said to uphold the belief that tomatoes, as food, are associated with cancer; but, on being interrogated, he flatly denies this, and expresses his personal fondness for the wholesome fruit, whether eaten with sugar or cooked as a vegetable. It should be noted that the cheap tomatoes sold by hucksters, though fleshy and well grown, are often unripe, with a lacklustre aspect which serves to condemn them as wanting the full flavour and the excellent virtues of the perfect fruit. Our American cousins, who are the enterprising fathers of this alterative Love Apple, persuade themselves that they are never in perfect health except during the tomato season. And, with ourselves, the ruddy solanum has obtained its popularity, not simply as a delicate, inviting vegetable product, but essentially as a stomachic, and a supposed purifier of the blood. In addition to the acids which have been named, the tomato furnishes a certain specific principle, which, when concentrated, brings about effects very similar to those which follow the administration of mercurial salts. They are an ulcerative condition of the mouth with a profuse flow of saliva, whilst the liver is stimulated irritatively to excessive action: peevishness also on the next day, with a depressing back ache in men, suggesting paralysis; and with a profuse fluor albus in women. When given moderately, as food or as physic, the fruit will remedy these same particular symptoms. By reason of its efficacy in promoting an increased flow of bile, when judiciously eaten, the tomato bears the name in America of "Vegetable Mercury," and it has almost superseded calomel there as a biliary medicinal agent. But some caution is needed against taking a surfeit of the fruit. Like other raw foods from the garden, tomatoes, if taken too freely, will irritate the intestines, especially by the mechanical presence of their seeds and skins. Several cases are on record of serious inflammation produced in the bowels by feeding exclusively for a while on uncooked tomatoes. *Per contra*, Dr. Griffith, in Australia, has given a fluid extract of the tomato in many cases of ulcerative sore mouth with marked success. Dr. Bennett, in this country, declares the tomato to be the most useful and the least harmful of all known medicaments for obviating derangements of the liver. He calls it a sovereign remedy for dyspepsia and bilious indigestion. Across the Atlantic a medicinal tincture is authoritatively made from the tomato for curative purposes by treating the apples and the bruised fresh plant with alcohol, and letting this stand for eight days before it is filtered and strained. A teaspoonful of the tincture is the proper dose, with a small wineglassful of cold water three or four times in the day. It may be that some curative notions attach themselves in the popular mind to the tomato because of its striking scarlet colour. Fabricius relates that as far back as in 1700 red plants were given for disorders of the blood, and yellow for those of the liver. The colour red was then regarded as representing heat, and was therefore thought to be in itself, after a manner, heat. A Japanese authority testifies as to the children of the royal house being laid, when sick, in chambers where the beds and the walls were covered with red, whilst all those who approached were clothed in scarlet. If this fact be in any degree true about tomatoes, they may be well supposed to fall short in curative excellence, and in fulness of flavour, unless thoroughly ripe, with perfect redness of colour. The taste for tomatoes is at first an acquired one, but with most persons it soon becomes lovingly confirmed. Spaniards and Italians eat them with pepper and oil. We also take them as a salad, or stewed with butter after they have been sliced and stuffed with bread crumbs and a spice of garlic. The whole fresh plant, if heated, emits an aroma which is acrid and malodorous; its vapour is so powerful as to sometimes cause giddiness and sickness. The late Mr. Shirley Hibberd, who was no mean naturalist, asserted, with seeming veracity, that the cannibal inhabitants of the Fiji Islands hold in high repute a native tomato, which is named by them the Solanum Anthropophagorum, and which they eat—"par excellence,"—with cold missionary.

